# Ultra-low concentration PVA-doped PMMA: an all-organic dielectric with markedly improved dielectric properties and energy storage performance

**DOI:** 10.1039/d5ra08529b

**Published:** 2026-02-05

**Authors:** Yuhao Chen, Guang Liu, Yang Cui, Chen Chen, Bocheng Wang, Han Chen, Taiquan Wu, Lifang Shen, Shubin Yan

**Affiliations:** a School of Electrical Engineering, Zhejiang University of Water Resources and Electric Power Hangzhou 310018 China lg@zuwe.edu.cn; b College of Mechanical and Electrical Engineering, China Jiliang University Hangzhou 310018 P.R. China; c Zhejiang-Belarus Joint Laboratory of Intelligent Equipment and System for Water Conservancy and Hydropower Safety Monitoring Zhejiang University of Water Resources and Electric Power Hangzhou 310018 P.R. China; d Institute of Water Sciences, Zhejiang University of Water Resources and Electric Power Hangzhou 310018 P.R. China

## Abstract

Polymer dielectrics are widely used in the fabrication of dielectric capacitors due to their excellent insulating properties. However, the relatively low dielectric constant (*ε*_r_) limits the energy storage density (*U*_d_) of polymer dielectrics. This study fabricated P(VA)MMA composite films by incorporating ultralow-content (<0.1 wt%) PVA into PMMA matrices. By systematically varying the PVA doping concentration (0.025–0.075 wt%), the *ε*_r_ and *U*_d_ were effectively optimized. Notably, the P(VA_1)MMA film with 0.025 wt% PVA doping concentration achieved a high *U*_d_ of 7.83 J cm^−3^ at 580 MV m^−1^ electric field, representing a 29.64% enhancement compared to pure PMMA film while maintaining an energy storage efficiency of 86.34%. This study successfully developed all-organic dielectric materials with significantly enhanced dielectric constant and energy storage properties at ultra-low doping concentrations, which holds important implications for the advancement of dielectric capacitors.

## Abstract 2

By incorporating a small amount of PVA into PMMA, the dielectric constant of the composite dielectric was significantly increased with only a slight rise in dielectric loss, ultimately yielding a desirable energy storage density.

## Graphical abstract

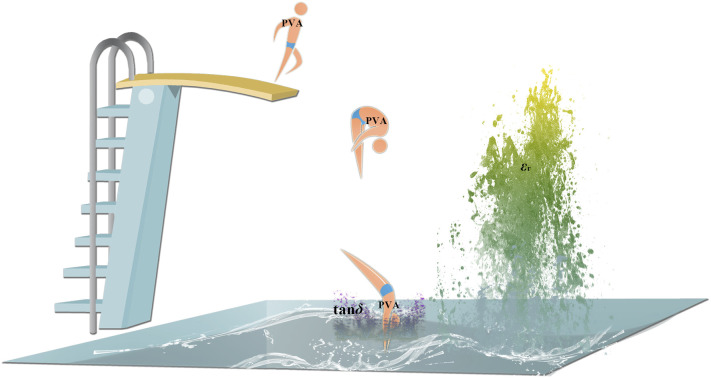

## Introduction

Compared to conventional energy storage devices such as batteries and electrochemical supercapacitors, dielectric capacitors have found widespread applications in microwave communications, pulsed power systems, power electronics, and distributed green energy generation, owing to their high power density and superior breakdown strength (*E*_b_).^1–3^ Based on material composition, dielectric capacitors can be classified into ceramic dielectric capacitors and polymer dielectric capacitors.^4^ Despite possessing high dielectric constant and excellent thermal stability, ceramic dielectrics are severely limited in flexible electronics and complex energy storage systems due to their intrinsically low *E*_b_ and poor mechanical flexibility.^5,6^ In contrast to ceramic materials, polymer dielectrics exhibit superior *E*_b_ and low dielectric loss tangent (tan*δ*), coupled with advantages such as light weight, facile processability, and excellent flexibility, demonstrating broad application prospects in dielectric capacitors.^7,8^ Generally, the energy storage density (*U*_d_) of polymer dielectrics can be calculated using eqn (1) and (2).1
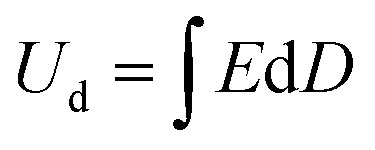

2 *D* = *ε*_0_*ε*_r_*E*

where *D* represents electric displacement polarization, *ε*_r_ denotes relative dielectric constant, and *ε*_0_ is the vacuum dielectric constant (8.85 × 10^−12^ F m^−1^).^9^ Therefore, the *ε*_r_ of polymer dielectrics directly governs their *U*_d_.^10^ In recent years, to enhance the dielectric constant of polymer dielectrics, significant research attention has been focused on constructing ceramic-polymer composite dielectrics by incorporating high-dielectric constant inorganic fillers (*e.g.*, BaTiO_3_, TiO_2_ NPs, NiCo_2_O_4_, MWCNTs) into either single-phase polymer matrices or multiphase polymer blends.^11–13^ However, this dielectric constant-enhancement strategy still faces several critical challenges. This approach requires high inorganic filler loadings, achieving improvement in dielectric properties at low filler loadings is typically the focus and goal of research on polymer nanocomposites.^14^ Additionally, the poor compatibility between inorganic fillers and the polymer matrix tends to induce particle clustering, resulting in increased leakage current and defect-mediated charge transfer, which consequently leads to a dramatic reduction in the composite's breakdown strength *E*_b_.^15–17^ Therefore, there is an urgent need to develop polymer materials with high *ε*_r_ that can significantly enhance the dielectric constant of dielectrics at low doping concentrations, thereby achieving a substantial improvement in *U*_d_.

Polymethyl methacrylate (PMMA) is an economically viable and practically useful polymer material, exhibiting outstanding mechanical strength, optical clarity, and chemical stability.^13^ As a classic linear polymer, PMMA has emerged as an ideal dielectric material for polymer-based capacitors due to its exceptionally high *E*_b_ and ultralow tan*δ*.^18,19^ However, its relatively low *ε*_r_ (∼5) severely limits the ability of PMMA to achieve superior *U*_d_.^20^ Polyvinyl alcohol (PVA), as a non-toxic polymer material, exhibits not only a high *ε*_r_ (>10) but also excellent biocompatibility, chemical stability, mechanical strength, and cost-effectiveness.^21,22^ More importantly, the hydroxyl groups (–OH) in PVA can interact with the electron-donating ester functionalities (O=C–O–CH_3_) in PMMA through hydrogen bonding networks, enabling effective polymer blending and facilitating the formation of stable, homogeneous composite materials.^23^ Therefore, doping PVA into PMMA represents an effective strategy for enhancing the dielectric constant of polymer composites. Moreover, while the PMMA/PVA polymer blend has been studied in fields such as biomaterials and optics, its application in the field of dielectric energy storage has not yet received much attention.

This study constructs a PVA/PMMA blend system using PMMA as the polymer matrix and PVA as the high-permittivity filler, with focused investigation on the dielectric constant, breakdown strength, and energy storage properties of the composite films at ultralow PVA doping concentrations (<0.1 wt%). The study reveals that doping PMMA with trace amounts of PVA significantly enhances the relative *ε*_r_ of the composite films. The composite films exhibit a *ε*_r_ approximately 1.24–1.37 times that of pure PMMA films. The study found that under identical electric field conditions, the composite films exhibit significantly enhanced *U*_d_ compared to pure PMMA films, while maintaining comparable charge–discharge efficiency (*η*). Notably, compared to the pure PMMA film, the energy storage density of the P(VA_1)MMA doped with 0.025 wt% PVA has been significantly improved. This work demonstrates an all-organic composite film fabricated by incorporating high-permittivity PVA into PMMA, which achieves significant enhancement in *ε*_r_ at ultralow doping concentrations, thereby boosting the *U*_d_. This strategy provides a novel paradigm for developing all-organic dielectric materials and holds important implications for advancing dielectric energy storage technologies.

## Experimental

### Materials

The fabrication of PVA/PMMA composite films required polymethyl methacrylate (PMMA) (Heat-resistant injection grade, Cat. No. P821343) and polyvinyl alcohol (PVA) (Mw ∼205 000, Cat. No. P119361), supplied by Shanghai Macklin Biochemical Co., Ltd and Shanghai Aladdin Biochemical Technology Co., Ltd, respectively. All chemical reagents and raw materials were used as received without further purification in the experimental procedures.

### Dielectric film fabrication

This study employed a solution casting method to fabricate PVA/PMMA composite films. First, PMMA pellets and *N*,*N*-dimethylformamide (DMF) solvent were precisely weighed at a ratio of 0.3 g mL^−1^. The PMMA pellets were added to the DMF solvent, and the mixture was continuously stirred at room temperature for 12 h to ensure complete dissolution of the PMMA pellets. Subsequently, PVA was weighed according to the target doping concentration and added to an appropriate amount of deionized water. The mixture was then left to stand at room temperature for 10 h until complete dissolution of PVA was achieved, yielding a saturated aqueous PVA solution. The saturated PVA aqueous solution was then dropwise added to the PMMA solution, followed by continuous stirring of the mixed solution at room temperature for over 12 h to ensure thorough blending of PMMA and PVA, ultimately yielding a stable homogeneous PVA/PMMA blend solution. Table 1 presents the mass fractions and corresponding gram weights of PVA in PVA/PMMA blend solutions with varying doping concentrations.

**Table 1:** The content of PVA in different P(VA)MMA composite films

Film abbreviations	Doping concentrations (wt%)	PVA (mg)
P(VA_1)MMA	0.025	0.350
P(VA_2)MMA	0.050	0.700
P(VA_3)MMA	0.075	1.050
P(VA_4)MMA	0.100	1.400

**Fig. 1:**
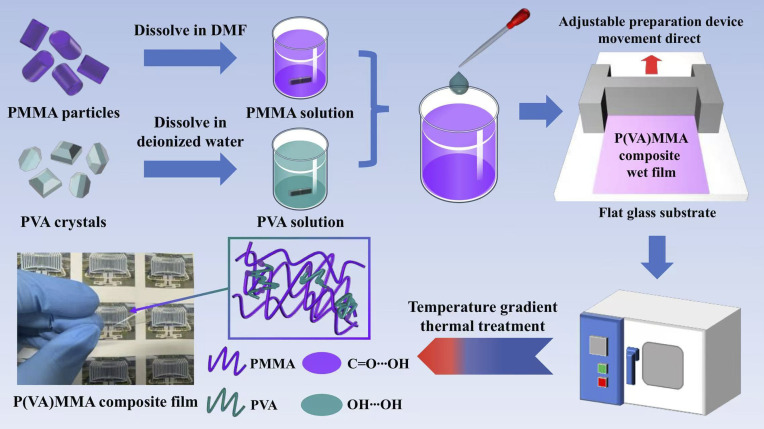
Fabrication process of P(VA)MMA composite films.

After degassing the PVA/PMMA blend solution in a vacuum drying oven, the homogeneous mixture was uniformly coated onto glass substrates using an automatic film applicator with an adjustable doctor blade, employing pristine flat glass plates as carriers. The glass substrates were then transferred to a temperature-programmed drying oven for temperature-gradient thermal treatment. The P(VA)MMA composite wet film was heated from 60 °C to 180 °C through a stepwise temperature ramp process (held at 60 °C for 6 h, 80 °C for 2 h, 100 °C for 2 h, 120 °C for 2 h, 150 °C for 2 h, and 180 °C for 2 h), with the entire procedure lasting approximately 16 h. Upon cooling to room temperature, the composite films were transferred to a vacuum oven and subjected to isothermal treatment at 120 °C under −0.1 MPa vacuum for 4 h, yielding the final P(VA)MMA composite films. The fabrication process of P(VA)MMA composite films is illustrated in Fig. 1. Additionally, as control groups, pure PMMA and PVA films were fabricated using identical processing protocols for comparative studies. This study experimentally observed that gelation occurs in PVA/PMMA blend solutions when the PVA doping concentration exceeds 0.1 wt%, resulting in a gel-like morphology as shown in SI Fig. S1. The gelled PVA/PMMA blend solution exhibits prohibitively high viscosity, precluding film fabrication *via* solution casting methods. Consequently, this study focused exclusively on comparative investigations of composite films with PVA contents below 0.1 wt%.

### Characterization and performance testing

In the experiment, the phase structure of the samples was characterized using a Bruker-D8 ADVANCE X-ray diffractometer, a Renishaw Invia confocal micro-Raman spectrometer, and an FTIR-650 Fourier transform infrared spectrometer. A ZEISS-GeminiSEM 360 scanning electron microscope was employed to observe the cross-sections of the samples to characterize their internal defects and thickness. The dielectric properties, breakdown electric field, and energy storage characteristics under different electric field strengths of the dielectric thin films were measured using a dielectric spectrometer (DMS1000 BALAB) and a ferroelectric tester (WGCM-20B Polyk). To prevent the adverse effects of moisture absorption on the performance tests, dehumidifiers and air conditioners were used to maintain the experimental humidity at below 40% RH in the long term. Additionally, prior to the aforementioned characterizations and performance tests, the samples were placed in a constant temperature drying oven at 60 °C for 12 hours to maintain dryness.

## Results and discussion

**Fig. 2:**
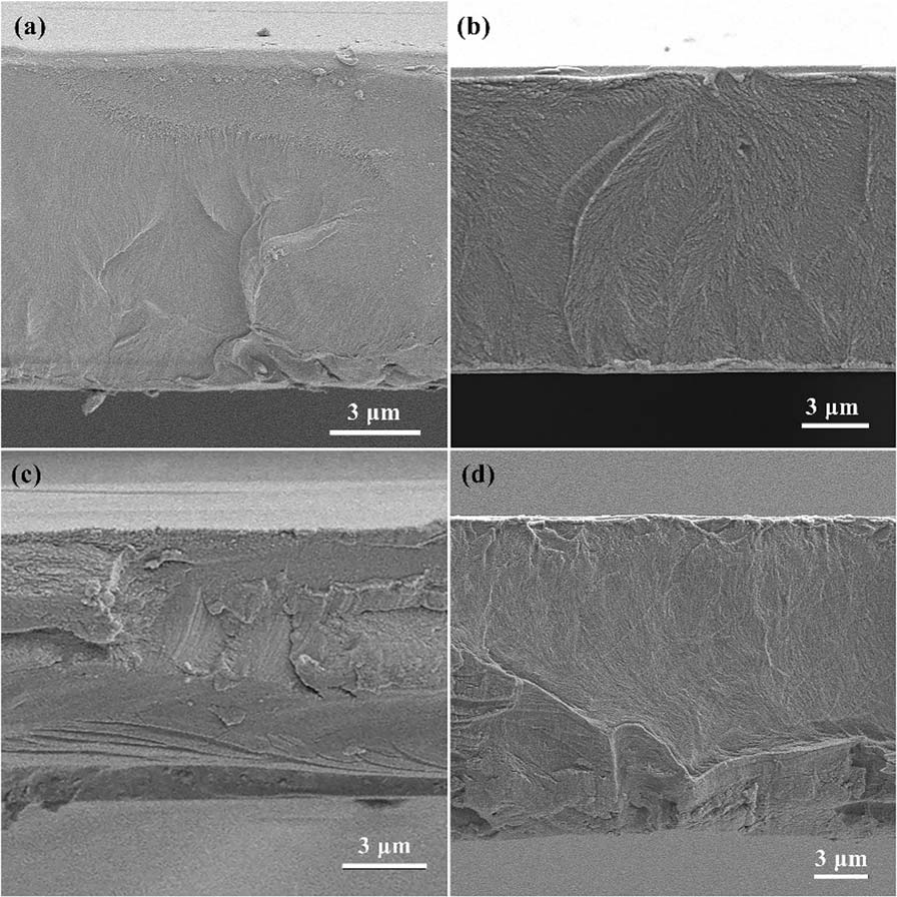
SEM image of the cross-section of the dielectric films. (a) PMMA; (b) P(VA_1)MMA; (c) P(VA_2)MMA and (d) P(VA_3)MMA.

To characterize the microstructural morphology of the dielectric films, the cross-section was characterized using scanning electron microscopy (SEM), with the results shown in Fig. 2. Evidently, neither pure PMMA films nor P(VA)MMA composite films with varying PVA doping concentrations exhibit discernible defects such as cracks or voids, demonstrating excellent compatibility between PVA and PMMA phases. Meanwhile, this defect-free morphology further confirms the high quality and structural integrity of the fabricated dielectric films.

**Fig. 3:**
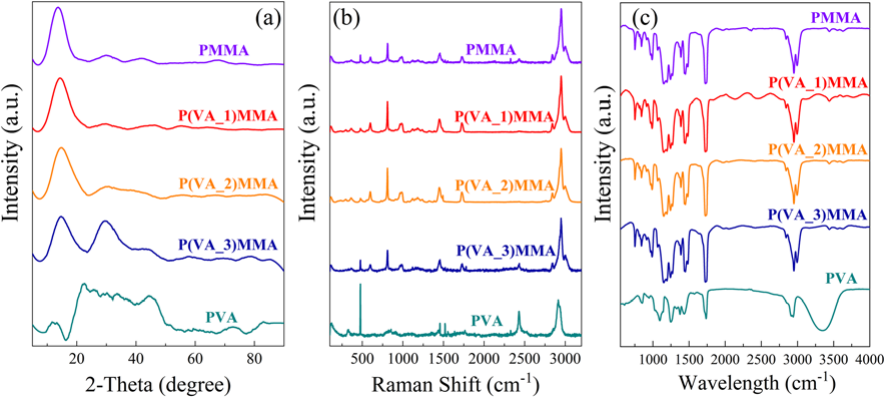
XRD, Raman and FTIR spectra of several dielectric films. (a) XRD; (b) Raman; (c) FTIR.

Fig. 3(a) presents the X-ray diffraction (XRD) patterns of pure PMMA, pure PVA, and P(VA)MMA composite films. The pure PMMA film exhibits broad amorphous halos centered at 14.53°, consistent with the non-crystalline characteristics of PMMA reported in prior studies.^24,25^ Previous studies have demonstrated that PVA typically exhibits an intense diffraction peak at 19.20°, accompanied by three weaker peaks at approximately 11.30°, 23.00°, and 41.00°.^26^ However, the XRD pattern of pure PVA film in Fig. 3(a) conspicuously lacks the characteristic intense peak at 19.20°, retaining only the three weaker peaks. This phenomenon arises because the temperature-gradient thermal treatment induces significant disruption of hydrogen-bonding networks in PVA, substantially diminishing the diffraction peak intensity at 19.20°.^26,27^ The XRD pattern of the P(VA)MMA composite film essentially coincides with that of pure PMMA, exhibiting identical broad amorphous halos at 14.53°, which confirms PMMA serves as the primary component in P(VA)MMA composite films and predominantly governs the crystalline structure of the composite films. Meanwhile, the P(VA)MMA composite films exhibits weak amorphous diffuse peaks near 30.06° and 41.68°, and the intensity of these diffuse peaks increases with the increasing doping concentration of PVA in the composite films. This indicates a superposition phenomenon between the diffuse peaks near 23.00° and 41.00° in PVA and those near 30.06° and 41.68° in PMMA, which fully confirms that the P(VA_1)MMA, P(VA_2)MMA, and P(VA_3)MMA films indeed contain PVA with progressively increasing doping concentrations as experimentally designed.^25,26^

The Raman spectra of pure PMMA, pure PVA, and P(VA)MMA composite films are presented in Fig. 3(b). The pure PMMA film exhibits a strong Raman peak at ∼2950 cm^−1^, flanked by two shoulder peaks at ∼2930 cm^−1^ and ∼3000 cm^−1^, along with a sharp peak near 800 cm^−1^ and five weaker peaks at approximately 1750 cm^−1^, 1450 cm^−1^, 970 cm^−1^, 600 cm^−1^, 500 cm^−1^, and 250 cm^−1^. This Raman profile is essentially consistent with previously reported PMMA spectra in the literature.^28^ The Raman peak at ∼2950 cm^−1^ originates from the symmetric C–H stretching vibration of CH_3_ groups, while the distinct shoulder peak at ∼2930 cm^−1^ arises from the O–CH_3_ stretching vibration.^29^ Theoretically, PVA should exhibit an intense Raman peak at ∼2830 cm^−1^ along with a series of weaker peaks in the 1000–1500 cm^−1^ spectral region.^30^ This series of weaker peaks corresponds to various vibration modes associated with the molecular structure of PVA, including CH_2_ wagging vibrations, C–H wagging vibrations, and CH out-of-plane bending vibrations.^31^

However, after the temperature-gradient thermal treatment, the pure PVA film retains only two weak peaks near 1500 cm^−1^, although the intense peak at ∼2830 cm^−1^ remains observable. This phenomenon similarly stems from the disruption of PVA's hydrogen-bonding networks by the temperature-gradient thermal treatment, thereby altering its molecular vibration modes. Evidently, the Raman spectral features of P(VA)MMA composite films closely correspond to those of pure PMMA films, confirming the dominant role of PMMA in the composite system and the absence of chemical reactions involving PMMA. When the PVA doping concentration is ≤0.05 wt%, the Raman peak intensities in the composite films exhibit a concentration-dependent enhancement with increasing PVA content. Conversely, when the PVA doping concentration exceeds 0.05 wt%, the composite films demonstrate a sharp reduction in Raman peak intensities. Simultaneously, the Raman peak intensities of P(VA_3)MMA films show remarkable similarity to those of pure PMMA films. This phenomenon can be attributed to PVA clustering, which creates large pure PMMA domains in P(VA_3)MMA films, making the PVA/PMMA interfaces undetectable by Raman spectroscopy.

To detect chemical solvent residues and moisture absorption in the dielectric films, FTIR measurements were performed to investigate the functional groups and chemical interactions of the films, as shown in Fig. 3(c). As shown in the figure, the transmission peaks of PMMA and the various P(VA)MMA samples exhibit almost no difference. The peak at 752.03 cm^−1^ represents the stretching vibration of –C–O–C–, while the peak near 990 cm^−1^ is attributed to the C–H rocking vibration.^32^ The stretching vibration of C–O–C results in another very strong characteristic peak of PMMA, appearing near 1140 cm^−1^. The peak at 1450 cm^−1^ represents the stretching vibration of the C=C group. At 1725 cm^−1^, a strong band originating from the carbonyl group (C=O) of PMMA can be observed. The stretching vibration of C–H leads to the characteristic band of PMMA appearing at 2940 cm^−1^. Most peaks of PVA overlap with those of PMMA, whereas the broad band at 3345 cm^−1^ is unique to PVA, representing the stretching vibration of –OH.^33^ The absence of this characteristic peak in both PMMA and PMMA-PVA indicates that the moisture absorption by the prepared dielectric films is not significant and can be neglected. Additionally, considering potential residues of the NMP solution, peaks representing the C–H stretching vibration of methyl groups and the C=O stretching vibration might be present.^34^ As can be seen from the figure, there are no significant changes in the C–H stretching vibration peaks and C=O stretching vibration peaks in PMMA and P(VA)MMA, which also suggests to a certain extent that there is virtually no NMP residue.

**Fig. 4:**
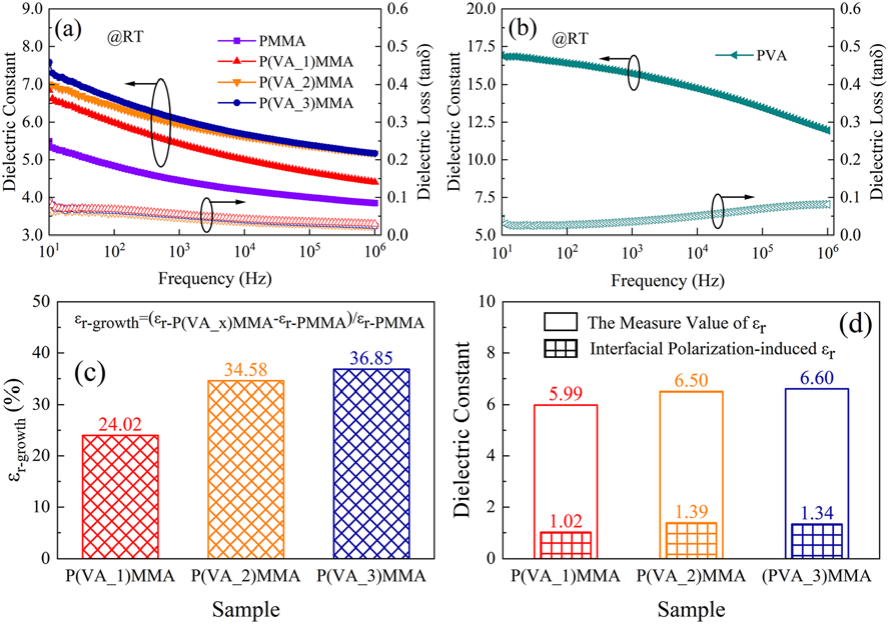
The dielectric properties of dielectric thin films. (a) Dielectric constant and loss *versus* frequency curves of dielectric films at RT; (b) dielectric constant and loss *versus* curves frequency of PVA at RT; (c) enhancement rate of *ε*_r_ for P(VA)MMA relative to PMMA at 100 Hz; (d) contribution of interfacial polarization to *ε*_r_ in P(VA)MMA at 100 Hz.

Initially, to investigate the dielectric properties of the thin films, the frequency-dependent dielectric constant (*ε*_r_) and loss tangent (tan*δ*) of dielectric films were measured at room temperature (30 °C) using an dielectric spectrometer, with the results presented in Fig. 4(a) and (b). At 100 Hz, the *ε*_r_ are measured as 4.83 for pure PMMA, 16.38 for pure PVA, 5.99 for P(VA_1)MMA, 6.50 for P(VA_2)MMA, and 6.61 for P(VA_3)MMA films. Evidently, the *ε*_r_ of P(VA)MMA composite films exhibits a progressive increase with elevating PVA doping concentrations. In the experiment, the PVA content added to PMMA is 0.025–0.075 wt%, which is only 1/200 or less of that used in most reported all-organic dielectric studies. For instance: adding 10 wt% of PNFA to PEI increased its dielectric constant only from 3.14 to 3.73 (@1000Hz); adding 10 wt% of FPE to PEI increased it only from 3.45 to 4.02 (@10Hz).^2,6^ However, even with such a trace amount of addition, the dielectric constant of PMMA was significantly increased from 5.49 to 7.58 (@10Hz), from 4.83 to 6.61 (@100Hz), and from 4.45 to 6.06 (@1000Hz). The magnitude of this enhancement in dielectric constant, achieved at an ultra-low loading, far exceeds that of the vast majority of reported research. Even at a low doping concentration of 0.025 wt%, the composite film achieves a 24.02% enhancement in *ε*_r_ compared to pure PMMA (as shown in Fig. 4(c)). This can be attributed to the hydrogen bonding network formed by the abundant –OH oligomers in PVA, along with the charge trapping effect at the PVA/PMMA interface.^23^ However, when the PVA doping concentration in P(VA)MMA composite films exceeds 0.05 wt%, further increases in PVA content yield diminishing returns in *ε*_r_ enhancement. This phenomenon occurs because PVA clustering at concentrations beyond this threshold reduces specific surface area, thereby limiting the expansion of PVA/PMMA interfacial area and consequently suppressing significant enhancement of interfacial polarization. Moreover, the *ε*_r_ of P(VA)MMA composite films exhibits strong frequency dependence, decreasing with rising frequency due to the inability of dipole polarization to keep pace with high-frequency electric fields.^2^ Concurrently, the tan*δ* of P(VA)MMA composite films remains comparable to that of pure PMMA despite increasing PVA doping concentrations. This achievement stems from both the ultralow doping levels (<0.1 wt%) and excellent PVA/PMMA compatibility, maintaining tan*δ* < 0.1 across the 10 Hz–10^6^ Hz frequency range. These dual factors collectively contribute to the stable low tan*δ* characteristics of P(VA)MMA composite films.

To further demonstrate the contribution of interfacial polarization within the composite material to the dielectric constant, the theoretical *ε*_r_ of the composite film at 100 Hz was calculated by combining eqn (3) and (4).^35^3
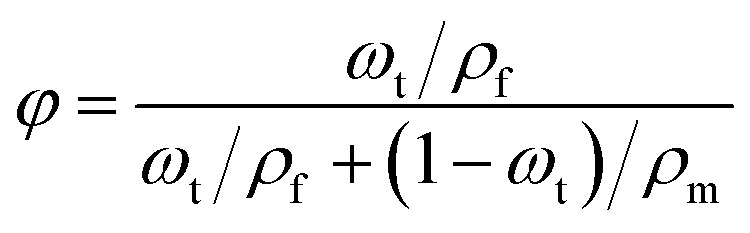


In this equation, *φ* represents the volume fraction of the dopant, *ω*_t_ the weight fraction, *ρ*_f_ the dopant density, and *ρ*_m_ the matrix density. Given the densities of PVA and PMMA as 1.27 g cm^−3^ and 1.19 g cm^−3^ respectively, the calculated PVA volume fractions are 2.34 vol%, 4.70 vol%, and 7.06 vol% for P(VA_1)MMA, P(VA_2)MMA, and P(VA_3)MMA films, respectively.

4 ln *ε*_c_ = *ϕ* ln *ε*_f_ + (1 − *ϕ*)ln *ε*_m_

**Fig. 5:**
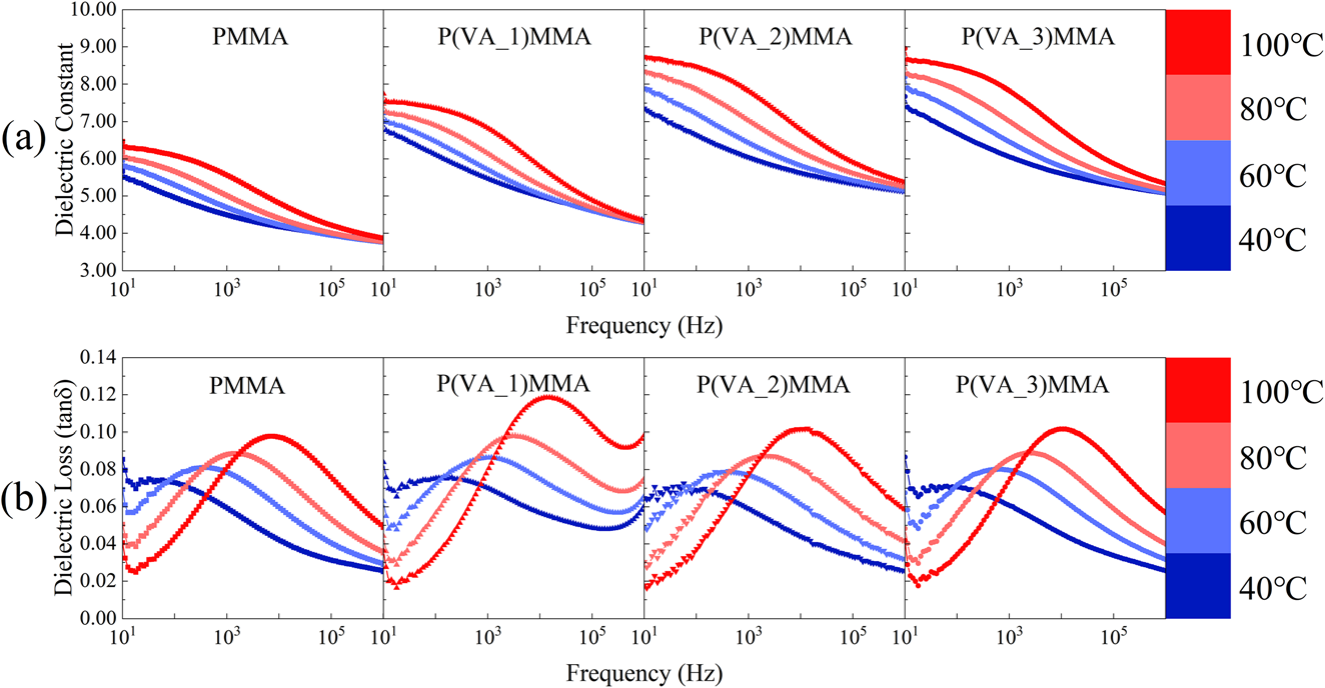
The dielectric properties *versus* frequency curves of dielectric films at different temperatures. (a) Dielectric constant; (b) dielectric.

In eqn (4), *ε*_c_, *ε*_f_, and *ε*_m_ represent the dielectric constants of the composite, dopant, and matrix, respectively. The comparison between the measured and theoretical values of *ε*_r_ for the composites at 100 Hz is shown in Fig. 4(d). Theoretically, the predicted *ε*_r_ values at 100 Hz for P(VA_1)MMA, P(VA_2)MMA, and P(VA_3)MMA films are 4.97, 5.11, and 5.26, which are significantly lower than the experimental results by margins of 1.02, 1.39, and 1.34, respectively. This discrepancy arises because the Lichtenecker logarithmic mixing rule (eqn (4)) does not account for interfacial polarization effects at PVA/PMMA interfaces.^36^ Consequently, the experimentally measured *ε*_r_ values of P(VA)MMA composite films consistently exceed those predicted by the Lichtenecker logarithmic mixing rule. This unequivocally demonstrates the critical role of interfacial polarization in enhancing the *ε*_r_ of P(VA)MMA composites. Based on the discrepancies between theoretical and experimental values, the interfacial polarization contribution rates to *ε*_r_ are calculated as 17.03%, 21.38%, and 20.30% for P(VA_1)MMA, P(VA_2)MMA, and P(VA_3)MMA films, respectively. It can be observed that when the PVA doping concentration exceeds 0.05 wt%, the contribution rate of interfacial polarization to *ε*_r_ decreases, demonstrating that PVA clustering reduces interfacial polarization in P(VA)MMA composites, thereby limiting further enhancement of *ε*_r_. With the increase in the doping concentration of polyvinyl alcohol (PVA), the *ε*_r_ of P(VA)MMA composite films exhibit a variation trend at high temperatures that is consistent with that room temperature, as shown in Fig. S2. However, due to molecular thermal relaxation, the tan*δ* of composite films increases with rising temperature, consequently degrading their charge–discharge efficiency (*η*) at elevated temperatures. Fig. 5(a) and (b) provides further analysis of temperature effects on the dielectric properties of both pure PMMA films and P(VA)MMA composite films. Starting from 40 °C, dielectric loss peaks emerge in the films, with these peaks shifting toward higher frequencies as temperature increases. Concurrently, the *ε*_r_ of the films gradually rises. This phenomenon occurs because the increase in temperature leads to enhanced carrier injection, which results in greater charge accumulation at the PVA/PMMA interface, thereby strengthening interfacial polarization.^7^

To investigate the breakdown characteristics of the dielectrics, the test samples are immersed in silicone oil to prevent surface flashover, and the voltage is ramped at a rate of 500 V s^−1^ until breakdown occurred. Eight independent samples were tested for each composition to ensure statistical significance. The measured breakdown values of the samples are listed in Table S1. The breakdown data were analyzed using the Weibull distribution function. The characteristic breakdown strength (*i.e.*, the strength at a 63.2% failure probability) and the Weibull shape parameter (*β*) were calculated according to the functional expression of the Weibull distribution:^8^

**Fig. 6:**
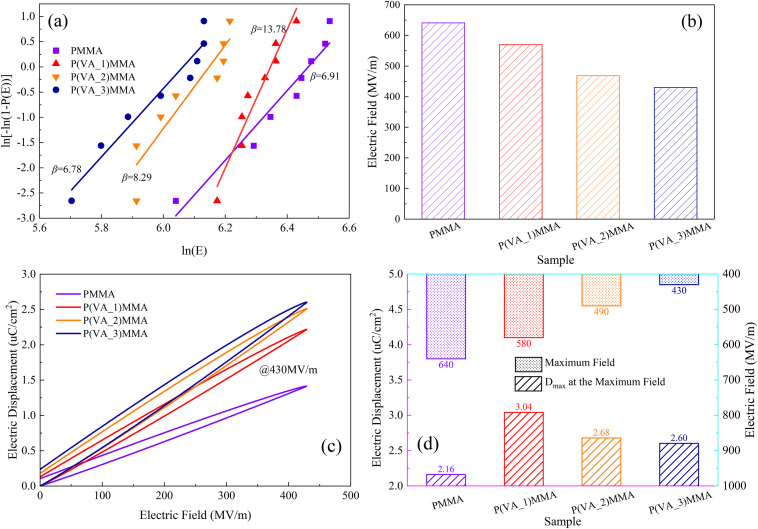
Breakdown characteristics and polarization characteristics of the dielectric thin films. (a) Weibull distribution; (b) characteristic breakdown electric field *E*_b_; (c) *D*–*E* curves of dielectric films under an electric field of 430 MV m^−1^; (d) maximum electric field intensities and corresponding maximum electric displacements of PMMA *versus* P(VA)MMA composite films.

5 *P*(*E*) = 1 − exp(−(*E*_*b*_/*E*_0_)^*β*^)

where *E*_b_ is the measured breakdown electric field of the sample, *E*_0_ represents the characteristic breakdown electric field at a cumulative failure probability of 63.2%. *β* is the shape parameter, which is used to measure the dispersion of the data points; a higher *β* value indicates better consistency of the breakdown electric field and greater reliability of the material. The Weibull distribution of the breakdown field strengths for several dielectric thin films and the corresponding *β* values are shown in the Fig. 6(a).

The statistical analysis reveals that the *β* values of the P(VA)MMA composite dielectrics are significantly higher than those of the pure PMMA films. This indicates that their breakdown processes possess excellent consistency and stability. Fig. 6(b) displays the characteristic breakdown electric field (*E*_0_) obtained from Weibull distribution statistics. The breakdown electric field of pure PMMA is significantly higher than that of the P(VA)MMA composite dielectrics, and its *E*_0_ shows a decreasing trend with increasing PVA content. The *E*_0_ value of P(VA_1)MMA (with a PVA content of 0.025 wt%) is 569.9 MV m^−1^, which is closest to the *E*_0_ value of PMMA (641.0 kV mm^−1^).

To further investigate the polarization characteristics of dielectric films, the electric displacement–electric field (*D*–*E*) curves of both pure PMMA and P(VA)MMA composite films were measured using a ferroelectric tester, with the corresponding results presented in Fig. S3. To ensure the reliability of the energy storage performance, we selected a set of measured data that is closest to the aforementioned characteristic breakdown electric field to plot the energy storage characteristics. As the applied electric field intensity gradually increases, the maximum electric displacement of the dielectric thin film progressively rises, while its *D*–*E* curve exhibits significant broadening. This indicates that the increase in electric field intensity deteriorates the charge–discharge efficiency (*η*) of the dielectric thin film. Among the composite films, the P(VA_3)MMA film exhibits the lowest withstandable electric field intensity, measuring only 430 MV m^−1^. To investigate the influence of PVA doping concentration on the polarization characteristics of P(VA)MMA composite films, the *D*–*E* curves of pure PMMA film and P(VA)MMA composite films under a 430 MV m^−1^ electric field were compared, as shown in Fig. 6(a). It is evident that the maximum electric displacement of P(VA)MMA composite films increases with higher PVA doping concentrations. However, when the PVA doping concentration exceeds 0.05 wt%, the rate of increase in maximum electric displacement significantly diminishes, which aligns with the variation trend of dielectric constant *versus* PVA doping concentration. Meanwhile, the *D*–*E* curves of P(VA)MMA composite films also exhibit pronounced broadening, indicating that increased PVA doping concentrations likewise deteriorate the *η* of the composite films.

Notably, the *D*–*E* curves width of P(VA_1)MMA film most closely approximates that of pure PMMA film, suggesting its potential for achieving higher *η*. Additionally, this study comparatively investigated the maximum withstandable electric field intensity and corresponding maximum electric displacement of pure PMMA films and P(VA)MMA composite films, as illustrated in Fig. 6(b). The pure PMMA, P(VA_1)MMA, P(VA_2)MMA, and P(VA_3)MMA films exhibited progressively decreasing maximum withstandable electric field intensities of 640 MV m^−1^, 580 MV m^−1^, 490 MV m^−1^, and 430 MV m^−1^, respectively. Under their respective maximum electric fields, the corresponding maximum electric displacements were measured as 2.16 µC cm^−2^, 3.05 µC cm^−2^, 2.68 µC cm^−2^, and 2.60 µC cm^−2^. It can be observed that the P(VA_1)MMA film exhibits only a 9.38% reduction in maximum withstandable electric field intensity compared to pure PMMA film, while achieving a remarkable 41.20% enhancement in maximum electric displacement. This significant improvement is attributed to the substantial increase in dielectric constant resulting from PVA incorporation, which establishes the foundation for P(VA_1)MMA film's superior energy storage density.

**Fig. 7:**
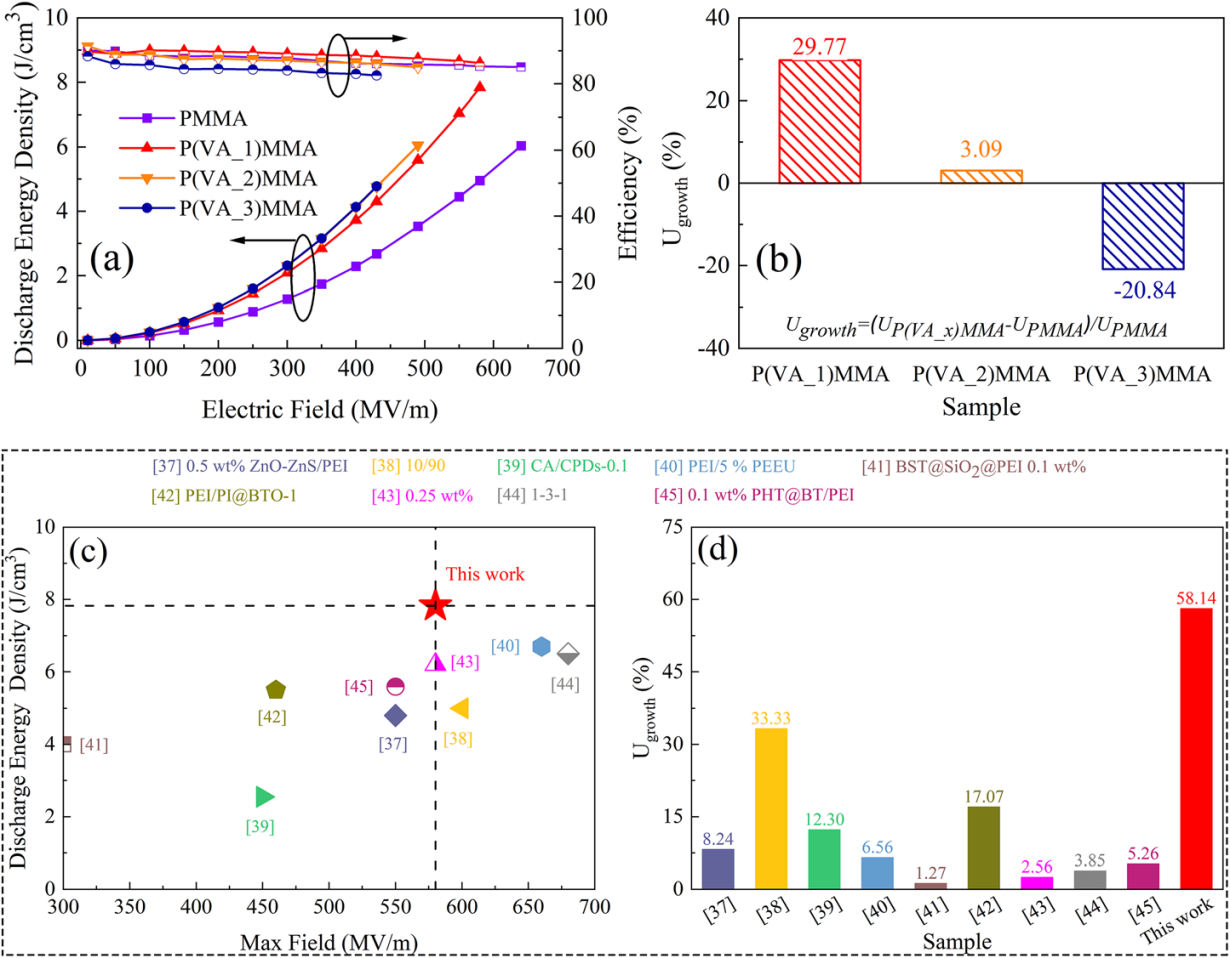
The energy storage characteristics of dielectric films. (a) *U*_d_ and *η* of dielectric films; (b) growth rate of *U*_d_ for P(VA)MMA composites relative to PMMA at their respective maximum electric fields; (c) comparison of *U*_d_ at maximum fields; (d) the enhancement rates of *U*_d_*versus* polymer matrices between P(VA_1)MMA and the research results in recent years.^39–45^

Based on the *D*–*E* curves of pure PMMA films and P(VA)MMA composite films under varying electric field intensities, the energy storage density (*U*_d_) and charge–discharge efficiency (*η*) of these dielectric films were calculated, with the computational results shown in Fig. 7(a). As evident from the figure, the *U*_d_ of P(VA)MMA composite films progressively increases with higher PVA doping concentrations under identical electric field conditions. This enhancement directly correlates with the concurrent improvement in dielectric constant observed in P(VA)MMA composites at elevated doping levels. However, the P(VA_2)MMA and P(VA_3)MMA films exhibit remarkably similar *U*_d_. This phenomenon occurs because although the P(VA_3)MMA film demonstrates a 3.85% higher maximum electric displacement than P(VA_2)MMA film under 430 MV m^−1^, resulting in greater charging energy density (*U*_c_) as shown in Fig. S4. However, the broadening of *D*–*E* curves in P(VA_3)MMA film leads to a 4.09% reduction in *η* compared to P(VA_2)MMA film under the same electric fields, consequently resulting in nearly identical *U*_d_ values between the two composite films. At their respective maximum electric field strengths, the pure PMMA film, P(VA_1)MMA film, P(VA_2)MMA film, and P(VA_3)MMA film exhibited *U*_d_ of 6.04 J cm^−3^, 7.83 J cm^−3^, 6.06 J cm^−3^, and 4.78 J cm^−3^, with corresponding *η* of 85.11%, 86.34%, 85.03%, and 82.57%, respectively. Based on these values, the growth rates of *U*_d_ in P(VA)MMA composite films relative to pure PMMA film were calculated at their maximum field strengths, as presented in Fig. 7(b). The P(VA_1)MMA and P(VA_2)MMA films demonstrated 29.77% and 3.09% enhancements in *U*_d_ respectively compared to pure PMMA film, while the P(VA_3)MMA film showed a 20.84% reduction in *U*_d_. Particularly noteworthy is that at an electric field strength of 580 MV m^−1^, the P(VA_1)MMA film achieved a remarkable 58.14% enhancement in *U*_d_ compared to pure PMMA film with only 0.025 wt% PVA doping.

Furthermore, even at their respective maximum field strengths, the P(VA_1)MMA film maintained a 29.64% *U*_d_ improvement over pure PMMA. Fig. 7(c) and (d) comparatively presents the maximum *U*_d_ of P(VA_1)MMA films *versus* high-dielectric filler-doped polymer systems from prior studies at low doping concentrations (<10 wt%), and the *U*_d_ enhancement rates of composites relative to pure polymermatrices under identical electric fields.^37–45^ It is evident that the P(VA_1)MMA film demonstrates significant advantages over current study in both doping concentration requirements and achievable energy storage density. The currently reported studies universally employ high-dielectric-material doping concentrations exceeding 0.1 wt%, whereas this work achieves exceptional performance with an ultra-low doping concentration of merely 0.025 wt%, representing a fourfold reduction compared to conventional systems. Remarkably, this all-organic composite film delivers a record-high energy storage density of 7.83 J cm^−3^ under such minimal doping conditions, demonstrating significant superiority over numerous inorganic-doped composite films with comparable low doping levels. Furthermore, the P(VA_1)MMA film exhibits the highest enhancement rate among all reported systems, demonstrating that ultra-low-concentration PVA doping can remarkably improve the energy storage properties of PMMA matrix. At its maximum withstandable electric field strength (580 MV m^−1^), the P(VA_1)MMA film achieves an outstanding *U*_d_ of 7.83 J cm^−3^ while maintaining an excellent *η* of 86.34%.

To further highlight the advantages of this study, we have supplemented the manuscript with a comparison of the energy storage density and charge–discharge efficiency of P(VA_1)MMA with previously reported energy storage dielectrics (doping content ≤1 wt%) at an electric field strength of 550 MV m^−1^, as shown in Table 2. As can be seen from the table, most reported studies dope high-permittivity inorganic materials into the polymer matrix. While this significantly increases the energy storage density at low doping levels, the charge–discharge efficiency is often unsatisfactory; although the charge–discharge efficiency can reach 90%, the energy storage density remains around 5 J cm^−3^. In comparison, the P(VA_1)MMA prepared in this work achieves an energy storage density of 7.04 J cm^−3^ with a PVA content as low as 0.025 wt% (far lower than most currently reported studies), while maintaining a charge–discharge efficiency of 86.99%. Thus, its comprehensive performance demonstrates significant advantages.

**Table 2:** Comparison of *U*_d_ and *η* between P(VA_1)MMA and nanocomposites in the references at an electric field strength of 550 MV m^−1^

Materimal	Content	*U*_d_(J cm^−3^)	*η*	Ref.
ZnO-ZnS/PEI	0.5 wt%	∼4.75	∼88%	37
BT/PEI	0.1 wt%	∼5.8	∼91%	45
PEI/BT@BN@AO	0.25 wt%	∼5	∼92%	46
TiO_2_/PESU	1 wt%	∼6	∼90%	47
0.1 wt% NH_2_-β-CD	0.1 wt%	∼4.5	∼90%	48
0.3 wt% NH_2_-β-CD	0.3 wt%	∼4.8	∼90%	
0.5 wt% NH_2_-β-CD	0.5 wt%	∼5	∼90%	
CD/PVDF	0.05 wt%	∼13.5	∼58%	49
BT@COF	0.1 wt%	∼14	∼55%	50
SnS_2_/BBPM	0.3 wt%	∼14	∼58%	51
P(VA_1)MMA	0.025 wt%	7.04	86.99%	—

## Conclusions

In summary, this work demonstrates a breakthrough in all-organic dielectric materials by employing PMMA as the matrix and PVA as high-dielectric filler, achieving significantly enhanced energy storage characteristics at remarkably low doping concentrations. By incorporating highly dielectric PVA into the PMMA matrix at an ultralow mass fraction of merely 0.025 wt%, this study leverages the interfacial polarization effect at PVA/PMMA interfaces to achieve a remarkable enhancement in the polymer film's dielectric constant while maintaining low dielectric loss and high charge–discharge efficiency. Ultimately, the P(VA_1)MMA film achieves a high energy storage density of 7.83 J cm^−3^ at 580 MV m^−1^, representing a significant increase of 2.88 J cm^−3^ compared to pure PMMA film, while maintaining an excellent charge–discharge efficiency of 86.34%. Compared with all the research results reported to date, the P(VA_1)MMA film exhibits excellent energy storage characteristics at an ultra-low doping concentration. This study pioneers a novel strategy for significantly enhancing the energy storage properties of all-organic dielectrics at ultralow doping concentrations, representing a major advancement for the energy storage field.

## Author contributions

Yuhao Chen: methodology, supervision, data curation, visualization and writing – original draft; Guang Liu: methodology, conceptualization, data curation, writing – review & editing and funding acquisition; Yang Cui: methodology, data curation and writing – review & editing; Chen Chen: methodology, visualization, data curation and funding acquisition; Bocheng Wang: conceptualization, validation, visualization and data curation; Han Chen: data curation, validation and writing – review & editing; Taiquan Wu: methodology and resources; Lifang Shen: methodology and resources; Shubin Yan: methodology, resources and funding acquisition.

## Conflicts of interest

There are no conflicts to declare.

## Supplementary Material

RA-016-D5RA08529B-s001

## Data Availability

All data generated or analysed during this study are included in this published article and its supplementary information (SI) files. Supplementary information: supporting data on dielectric properties, *D*–*E* hysteresis loops, and charge–discharge energy densities. See DOI: https://doi.org/10.1039/d5ra08529b.
