# Supplementary material for: Ultra-low concentration PVA-doped PMMA: an all-organic dielectric with markedly improved dielectric properties and energy storage performance
Source: RSC Adv. 2026 Feb 5;16(8):7618–27. doi: 10.1039/d5ra08529b (PMC12875313; doi:10.1039/d5ra08529b)
Supplement: RA-016-D5RA08529B-s001 [file RA-016-D5RA08529B-s001.pdf]

Supplementary Information (SI) for RSC Advances.
This journal is © The Royal Society of Chemistry 2026

Supplementary Information

Ultra-low concentration PVA-doped PMMA: An all-organic dielectric with markedly improved dielectric properties and energy storage performance

Yuhao Chen[a], Guang Liu[a,c]*, Yang Cui[a,b,c], Chen Chen[a,b,c], Bocheng Wang[a,b], Han Chen[a,b], Taiquan Wu[a,c,d], Lifang Shen[a], Shubin yan[b,c,d]

[a] *School of Electrical Engineering, Zhejiang University of Water Resources and Electric Power, Hangzhou 310018, China.*

[b] *College of Mechanical and Electrical Engineering, China Jiliang University, Hangzhou 310018, P.R. China.*

[c] *Zhejiang-Belarus Joint Laboratory of Intelligent Equipment and System for Water Conservancy and Hydropower Safety Monitoring Zhejiang University of Water Resources and Electric Power, Hangzhou 310018, P.R. China.*

[d] *Institute of Water Sciences, Zhejiang University of Water Resources and Electric Power, Hangzhou 310018, P.R. China.*

* Corresponding author.

E-mail address: lg@zuwe.edu.cn.

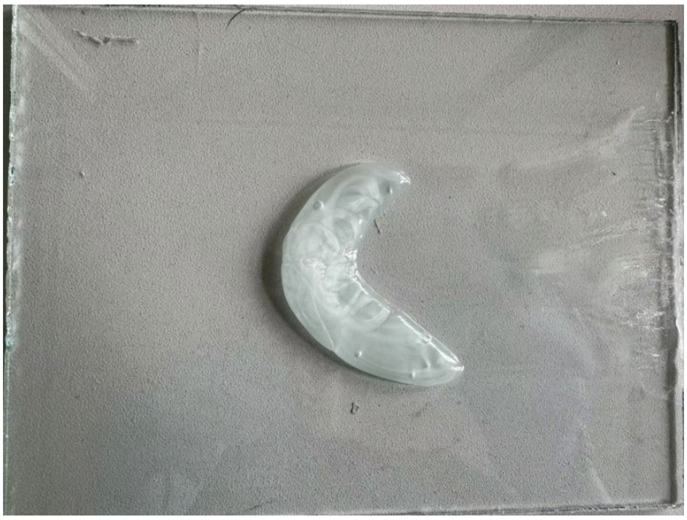

Fig. S1. The PVA/PMMA blended solution undergoes gelation transformation.

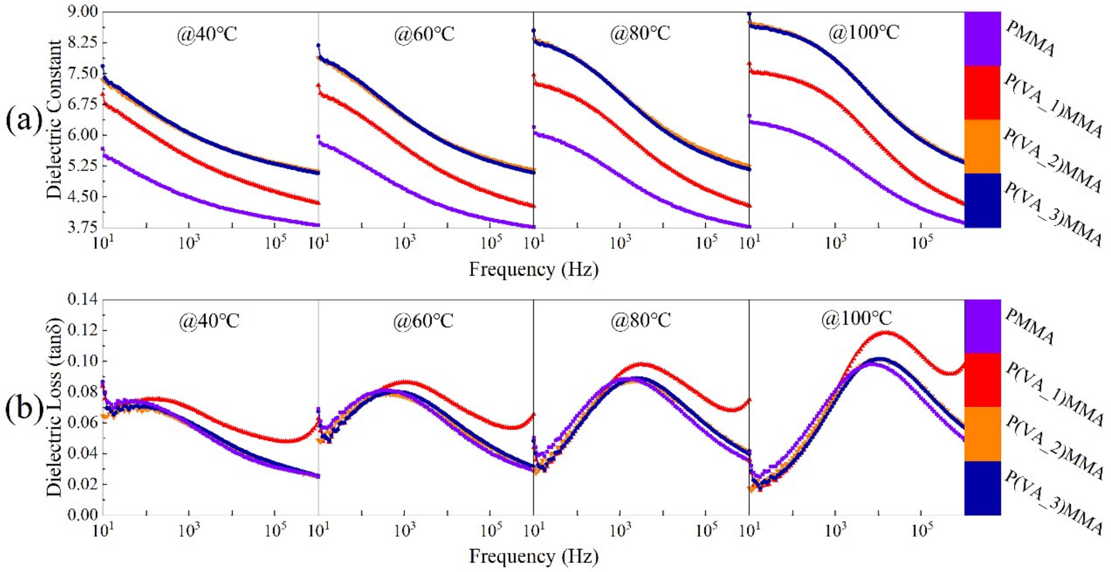

Fig. S2. (a) Dielectric constant versus frequency and (b) Dielectric loss versus frequency curves of pure PMMA films and P(VA)MMA composite films at different testing temperatures

Fig. S2 demonstrates the frequency-dependent dielectric constant (ε_r) and dielectric loss tangent (tanδ) of pure PMMA films and P(VA)MMA composite films at varying test temperatures (40°C, 60°C, 80°C, 100°C).

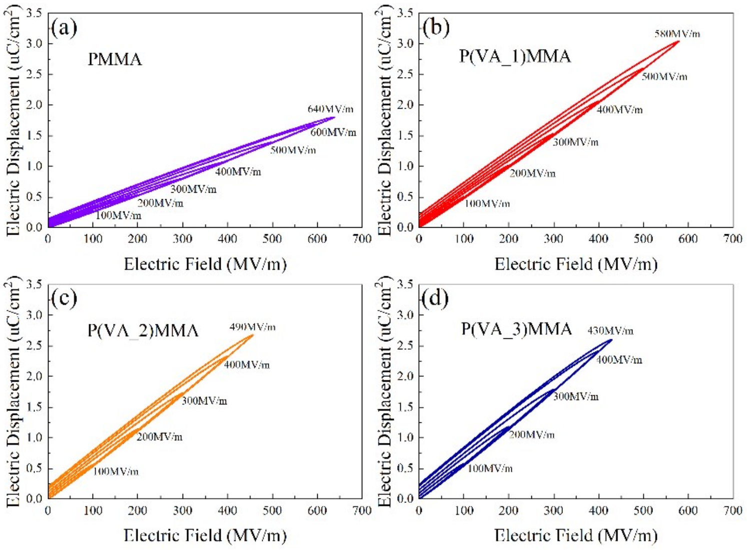

Fig. S3. D-E curves of pure PMMA films and P(VA)MMA composite films under different electric field intensities

Figs. S3 (a)-(d) respectively present the D-E curves of pure PMMA, P(VA_1)MMA, P(VA_2)MMA, and P(VA_3)MMA films under varying electric fields.

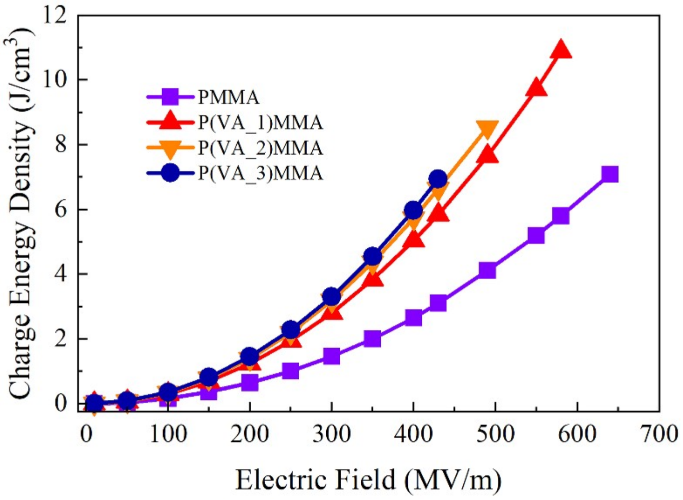

Fig. S4. U_c of pure PMMA films and P(VA)MMA composite films

Fig. S4 presents the charging energy density (U_c) of pure PMMA, P(VA_1)MMA,

P(VA_2)MMA, and P(VA_3)MMA films.

Table S1. The measured breakdown electric field (E_b) values of the dielectric thin films.

Materimal	E_b (MV/m)							
	1	2	3	4	5	6	7	8
PMMA	420	540	570	620	630	650	680	690
P(VA_1)MMA	480	520	520	530	560	580	580	620
P(VA_2)MMA	370	370	400	420	480	490	490	500
P(VA_3)MMA	300	330	360	400	440	450	460	460
